# Medical Items

**Published:** 1912-11

**Authors:** 


### MEDICAL ITEMS

### AFTER SCARLET FEVER AND MEASLES.

After the acute diseases of childhood there is no remedy that will do more to hasten convalescence than Gray's Glycerine Tonic Comp. Children are particularly responsive to the tonic effects of "Gray's" and it is always gratifying to see the prompt improvement in the appetite, digestion and general nutrition that follows its administration. The palatability and clean bitter taste of "Gray's" make it exceptionally acceptable to children.

